# The Impact of Pre-Exposure Prophylaxis (PrEP) on HIV Epidemics in Africa and India: A Simulation Study

**DOI:** 10.1371/journal.pone.0002077

**Published:** 2008-05-07

**Authors:** Debby C. J. Vissers, Hélène A. C. M. Voeten, Nico J. D. Nagelkerke, J. Dik F. Habbema, Sake J. de Vlas

**Affiliations:** 1 Department of Public Health, Erasmus MC, University Medical Center Rotterdam, Rotterdam, The Netherlands; 2 Department of Community Medicine, United Arab Emirates University, Al-Ain, United Arab Emirates; University of Cape Town, South Africa

## Abstract

**Background:**

Pre-exposure prophylaxis (PrEP) is a promising new HIV prevention method, especially for women. An urgent demand for implementation of PrEP is expected at the moment efficacy has been demonstrated in clinical trials. We explored the long-term impact of PrEP on HIV transmission in different HIV epidemics.

**Methodology/Principal Findings:**

We used a mathematical model that distinguishes the general population, sex workers and their clients. PrEP scenarios varying in effectiveness, coverage and target group were modeled in the epidemiological settings of Botswana, Nyanza Province in Kenya, and Southern India. We also studied the effect of condom addition or condom substitution during PrEP use. Main outcome was number of HIV infections averted over ten years of PrEP use. PrEP strategies with high effectiveness and high coverage can have a substantial impact in African settings. In Southern India, by contrast, the number of averted HIV infections in different PrEP scenarios would be much lower. The impact of PrEP may be strongly diminished or even reversed by behavioral disinhibition, especially in scenarios with low coverage and low effectiveness. However, additional condom use during low coverage and low effective PrEP doubled the amount of averted HIV infections.

**Conclusions/Significance:**

The public health impact of PrEP can be substantial. However, this impact may be diminished, or even reversed, by changes in risk behavior. Implementation of PrEP strategies should therefore come on top of current condom campaigns, not as a substitution.

## Introduction

Behavioral changes, such as reduction in the number of sex partners and the use of barrier methods in high-risk contacts, have slowed down the HIV epidemic in many places in the world [1], [2] and will be of importance as long as no vaccine is available. Condom use, the main barrier method, is mainly male-controlled. Although condoms could, potentially, stop sexual HIV transmission almost completely, new intervention strategies are still urgently needed, especially those that can help women protect themselves. A recent microbicides trial testing cellulose sulphate was stopped prematurely because the gel was not only ineffective, but actually increased HIV risk [3], [4]. Furthermore, recent trials with a diaphragm intervention method or with an HIV vaccine did not show any benefit [5], [6]. Pre-exposure prophylaxis (PrEP) seems a promising new intervention [7]–[9] to fill the gap in female-controlled prevention, but the method may be equally effective for males.

PrEP means that HIV-negative people regularly take antiretroviral (ARV) drugs to prevent infection [8], [10], [11]. The concept of using ARV as a preventive method has been tested and proven successful in prevention of mother-to-child transmission of HIV [12]. Perhaps more significantly, post-exposure prophylaxis (PEP) in health care workers immediately after accidental exposure to HIV is common practice and may prevent 80% of the HIV infections due to needle accidents [13].

Animal HIV challenge studies provided preliminary evidence that PrEP might be partially effective in preventing HIV infection [14]–[16]. The ARV drug tenofovir prevented simian immunodeficiency virus (SIV) infection in macaques, when given 48 hours before an intravenous exposure to SIV [14]. Yet, while delaying SIV infection, tenofovir could not fully prevent infection after repeated viral challenges [15]. A combination of tenofovir and emtricitabine (FTC) however provided a high level of protection in humanized BLT mice [16].

To determine safety and efficacy of tenofovir and tenofovir/FTC in humans, clinical trials are currently ongoing in young adults (Botswana), injection drug users (Thailand), and men who have sex with men (United States, Peru/Ecuador) [17]. Preliminary results of a phase II safety trial among female sex workers in Ghana, Nigeria and Cameroon showed that the use of tenofovir was not associated with adverse events [18]. However, efficacy could not be determined due to the low number of HIV infections. Other trial results are expected at the earliest in 2008–2009 [17], [19].

Some fear that the use of PrEP may lead to more risky sexual behavior because people may feel protected against HIV infection [20], [21]. This increase, which is called behavioral disinhibition or risk compensation [22], would to some extent reduce the effect of PrEP. Persons taking PrEP may feel protected against HIV infection and consequently use fewer condoms. On the other hand, PrEP users may be extensively counselled, be more aware of their risk behaviour and the risks of unprotected sex, and may therefore be more likely to use condoms. Nevertheless, whether or not PrEP will lead to changes in risk behavior remains uncertain especially if upscaling of services would lead to less effective counselling.

PrEP will directly protect individuals taking it, but may also have an indirect effect on non-PrEP users since reduced numbers of HIV infections will lead to decreased transmission. Mathematical models can be used to estimate these indirect effects. Accurate projections of the effect of PrEP on populations may help policy makers in their decision process and planning of PrEP services in AIDS control programmes. A demand for implementation of such programmes is expected at the moment efficacy of tenofovir or tenofovir/FTC against HIV transmission is proven in the different clinical trials [19]. We used a mathematical model that has previously been used to study the effect of HIV vaccines and male circumcision [23], [24]. In this study, we examined the long-term effect of different levels of PrEP effectiveness on HIV transmission in populations differing in HIV epidemiology.

## Methods

### HIV model

We adapted an existing compartmental HIV transmission model to study the impact of PrEP on HIV epidemics in three different regions, namely Botswana, Nyanza Province in Kenya and Southern India [23], [24]. This model divides the population in groups of low-risk persons (not involved in sex work) and high-risk persons (male clients and female sex workers), further subdivided into compartments by HIV infection status, stage of infection, and PrEP use. HIV infection was defined as early in the first four years of infection and as late in the last four years. Per gender and risk group, five compartments are distinguished in our model: HIV-negative not using PrEP; HIV-negative using PrEP; HIV-early not using PrEP; HIV-early using PrEP; and HIV-late not using PrEP. AIDS and death are endpoints of the model.

Low-risk persons can become high-risk persons, and vice versa. We only modeled heterosexual transmission. HIV can spread from infected to uninfected persons by three relationship types. First, HIV transmission can occur in sexual contacts between female sex workers and their clients. Second, transmission can occur through marriage-like relationships. Third, “leakage” from infected individuals can occur reflecting all non-paid casual sex. HIV transmission through marriage and “leakage” only occurs in low-risk groups. Condom use is assumed in client-sex worker contacts, but not in other types of sexual relationships.

All other assumptions underlying the original model can be found elsewhere [23], [24]. A technical description of the model including compartments, flows, variables and parameters can be found in the Appendix S1 and Figure S1. ModelMaker® (version 3.0.3) was used to implement and run the model.

Parameter values to model HIV epidemics in Botswana and Nyanza were based on recent modeling work of Nagelkerke *et al*. [24], who explored the effect of male circumcision. In our model, circumcision was not taken into account. We slightly lowered the former female-to-male transmission risk to adjust for the protective effect of male circumcision. In earlier modeling work, condom use in commercial sex was set at 20%. Data of Nyanza province from 1999 showed that 34–56% of clients and 75% of sex workers reported that they always or usually used condoms during commercial sex [25]. Therefore, we assumed that condom use in commercial sex increased to 50% from 2000 onwards. All other parameter values were kept identical to earlier values [24]. The main difference between Botswana and Nyanza was the higher “leakage” in Botswana, reflecting an assumed higher number of casual contacts in this country (see Appendix).

These parameter choices yielded approximate equilibrium HIV prevalence levels of 33% in Botswana and 16% in Nyanza. In Botswana, as the actual reported national HIV prevalence was 24% in 2005 [26], our model therefore probably only reflects the worst affected parts of the country. In Nyanza, HIV prevalence was estimated at 15% in 2003 [27], similar to the model's prediction.

In India, the southern states are the most affected area [28]. Condom use during commercial sex is more than 85% since 2000 [29]. We assumed that condom use in commercial sex was 60% in 1994 and increased to 90% from 1998 onwards. Besides the level of condom use, the main difference between Botswana/Nyanza and Southern India was a lower “leakage”, reflecting an assumed lower frequency of casual contacts in India (see Appendix).

Our modeled HIV prevalence in India was based on two data sources. First, we used antenatal clinic (ANC) sentinel surveillance data from Southern India, which showed a decline in HIV prevalence from 1.8% in 1998 to 1.3% in 2004 [30], [31]. Second, we used a large, representative population based prevalence survey in a rural area in South India which found an overall prevalence of 2.9% in 2003 [32]. Because the latter study had a larger sample size and included the whole population instead of only pregnant women, it might be more representative for Southern India than the ANC data. Therefore, we simulated the decreasing trend of ANC data, but ended slightly higher at a 1.3% prevalence in 2007.

### Modeling PrEP

Only uninfected persons start taking PrEP. HIV-negatives can be identified by either Client-Initiated Testing and Counselling (CITC, formerly known as Voluntary Counselling and Testing) or Provider-Initiated Testing and Counselling (PITC) [33]. The coverage of PrEP is based on HIV-negative persons taking PrEP. Some will stop taking pills (non-adherence). Since PrEP will most likely not fully protect against HIV infection, persons may still get infected, but at a lower rate than those not on PrEP. We assumed that persons taking PrEP pills who get HIV infected, continue to use PrEP on average for one year.

The use of a single drug, such as tenofovir, could promote HIV resistance. However, a modeling study of the Botswana trial estimated that of 600 participants receiving tenofovir, 45 persons would seroconvert and less than one participant was expected to acquire or develop a resistant HIV strain (Smith *et al*., 2006, THAX0105–Antiretroviral resistance is not an important risk of the oral tenofovir prophylaxis trial in Botswana: a simple mathematical modelling approach, XVI International AIDS Conference, Toronto, Canada). Therefore, we assumed that taking PrEP pills will not lead to any resistance.

We assumed that PrEP would become available in 2012. We predicted the effect of two strategies: targeting only sex workers or targeting both sex workers and clients. This latter strategy resembles a general population intervention with high-risk individuals coming to clinics for PrEP. The efficacy of PrEP is not known yet. We thus, somewhat arbitrarily, assumed a 50% or 90% effectiveness (i.e. reduced HIV susceptibility of those taking PrEP). Coverages of persons taking PrEP varied per targeting strategy. For Botswana/Nyanza, we explored a low (25%) or high (75%) coverage. For Southern India, we assumed that targeting with PrEP would result in higher coverage rates, since condom use is also very high in commercial sex [29]. As taking one pill per day might be as easy, or easier, than using a condom in each sex act, we assumed that PrEP coverage rates in Southern India were 50% and 95%, respectively.

We supposed that when sex workers and clients who use PrEP pills stop their high-risk behaviour and become low-risk individuals, they will also stop taking PrEP pills (i.e. they are no longer part of the target group). Main outcomes were HIV prevalence, number of HIV infections averted over ten years of PrEP use (i.e. in 2022), and amount of PrEP needed in ten years. Both averted infections and amount of PrEP were calculated per 100,000 HIV-negative adult person years.

To explore alternatives to PrEP, we also modelled an intervention resulting in increased condom use in client-sex worker contacts. In this intervention, condom use was assumed to increase in 2007 (and not in 2012 like the PrEP strategies, since it is already available) from 50% to 75% in Botswana/Nyanza, and from 90% to 95% in Southern India. Thus, in both situations we assumed that the number of non-users was halved. Furthermore, we explored the effect of less (i.e. condom substitution) or more (i.e. condom addition) condom use during PrEP interventions. In substitution scenarios, the level of condom use was assumed to be 15% lower, 35% in Botswana/Nyanza and 75% in India (i.e. halfway the levels in 2007 and 2000 or 1998, respectively). In addition scenarios, condom use was assumed to be halfway that of the condom intervention level, 62.5% in Botswana/Nyanza and 92.5% in India.

## Results

**Figure 1:**
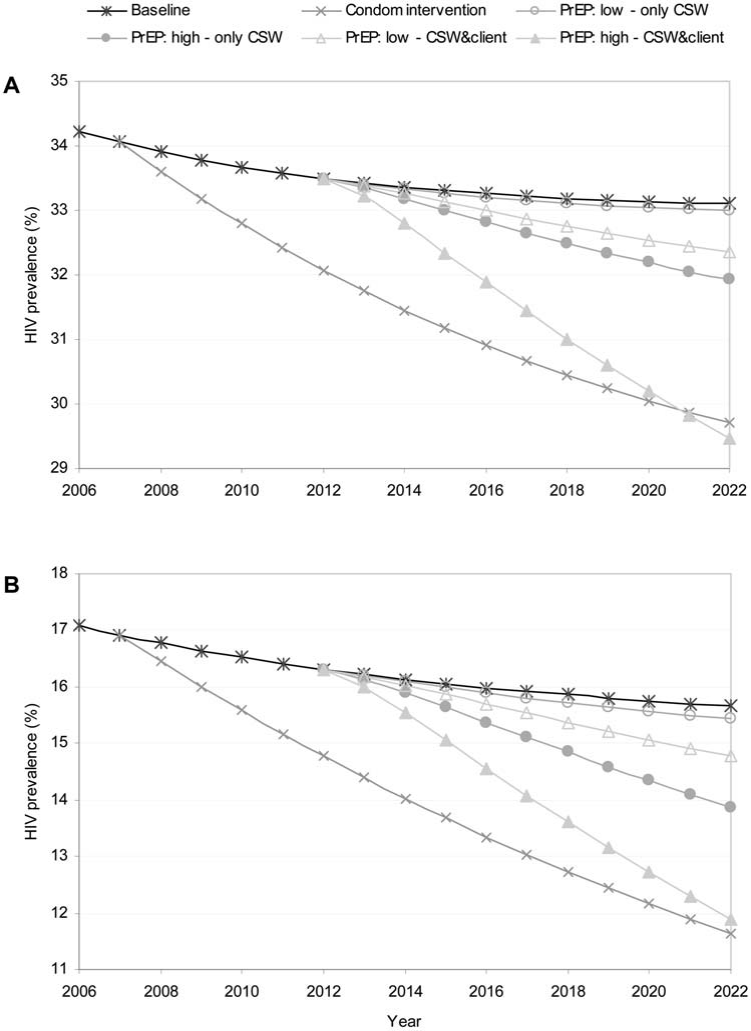
Effect of different PrEP scenarios and condom use on HIV prevalence. A: Botswana, B: Nyanza province, Kenya. ‘PrEP low’ means 25% coverage and 50% effectiveness; ‘PrEP high’ means 75% coverage and 90% effectiveness; ‘Only CSW’ means target group is sex workers; ‘CSW&client’ means target group is sex workers and clients.

The impact of different PrEP strategies and the condom intervention on HIV prevalence in Botswana and Nyanza over the period 2006–2022 is shown in Figure 1. PrEP strategies with high coverage and high effectiveness targeting only sex workers or both sex workers and clients have a substantial impact on HIV prevalence. In both settings, the condom intervention and the high PrEP scenario targeting both sex workers and clients resulted in comparable HIV prevalences in 2022.

**Figure 2:**
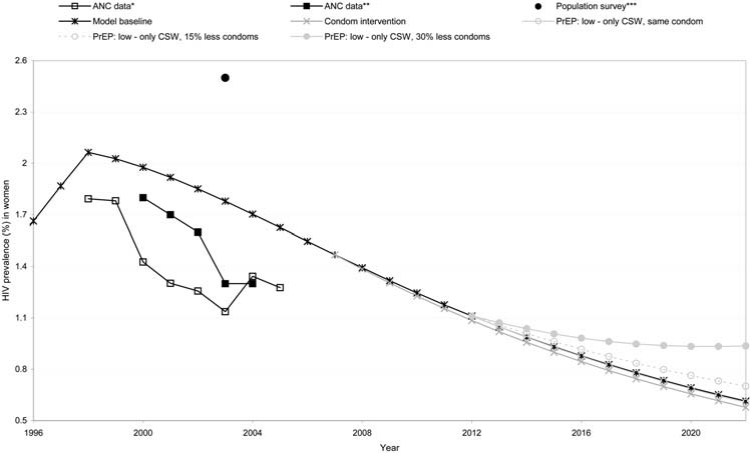
HIV prevalence in women in Southern India. Depicted are the baseline fit and the effect of different PrEP scenarios and condom use. *NACO, 2005 [30]; **Kumar *et al.*, 2006 [31]; ***Becker *et al.*, 2007 [32]. ‘PrEP low’ means 50% coverage and 50% effectiveness; ‘Only CSW’ means target group is sex workers; ‘Same’ means unchanged condom use during PrEP (90%); ‘Less’ means condom substitution during PrEP (condom use is 75% or 60%).

Figure 2 shows the baseline fit in Southern India. A PrEP scenario with 50% coverage and 50% effectiveness targeting only sex workers with three different condom options is also depicted. Condom substitution (i.e. 15% less use) during the PrEP scenario, resulted in a higher HIV prevalence, however, the prevalence was still decreasing. HIV prevalence no longer decreased if condom use during PrEP was reduced to 60% (i.e. 30% less use).

Results of different PrEP strategies and condom substitution or condom addition during PrEP are given in Table 1. The number of infections averted varied from 26 to 785 per 100,000 uninfected adult person years in Botswana, and from 44 to 733 per 100,000 person years in Nyanza. The number of averted infections was, even with higher coverages, considerably lower in Southern India: only 0.9 to 6.0 averted infections per 100,000 person years. The amount of PrEP pills needed in a 10-year period varied from around 50,000 to around 2 million pills per 100,000 person years in the African settings. Slightly more PrEP pills were needed in Southern India (Table 1).

**Table 1:** Impact of different PrEP scenarios in Botswana, Nyanza and Southern India.

Setting–PrEP scenarios^a^	PrEP efficacy (%)	PrEP coverage (%)	PrEP pills^c^ (×10^6^)	Averted HIV cases^d^ in case condom use was:		
				Same 50/90%^e^	Less 35/75%^e^	More 62.5/92.5%^e^
Botswana (N = 516,000^b^)						
Low–sex workers	50	25	0.04	26	−221	288
High–sex workers	90	75	0.18	251	−14	503
Low–sex workers/clients	50	25	0.83	159	−80	403
High–sex workers/clients	90	75	2.11	785	640	909
Nyanza (N = 837,000^b^)						
Low–sex workers	50	25	0.06	44	−236	325
High–sex workers	90	75	0.23	342	75	564
Low–sex workers/clients	50	25	0.77	166	−100	419
High–sex workers/clients	90	75	1.92	733	610	831
Southern India (N = 235,000,000^b^)						
Low–sex workers	50	50	0.25	0.9	−16.5	2.7
High–sex workers	90	95	0.51	3.8	−2.2	4.6
Low–sex workers/clients	50	50	1.50	1.8	−11.4	3.3
High–sex workers/clients	90	95	2.66	6.0	4.4	6.2

a All PrEP scenarios started in 2012. ‘Low’ and ‘high’ refer to PrEP effectiveness and PrEP coverage; ‘sex workers’ and ‘sex workers/clients’ refer to the different target groups.

b Adult population size in 2012.

c Number of PrEP pills per 100,000 uninfected adult person years needed in the period 2013–2022 in scenarios in which condom use was unchanged.

d Number of averted HIV infections per 100,000 uninfected adult person years in the period 2013–2022.

e ‘Same’ means unchanged condom use during PrEP: 50% in Botswana/Nyanza and 90% in Southern India; ‘Less’ means condom substitution during PrEP (i.e. less condom use): 35% in Botswana/Nyanza and 75% in Southern India; ‘More’ means condom addition during PrEP (i.e. more condom use): 62.5% in Botswana/Nyanza and 92.5% in Southern India.

Condom substitution (i.e. 15% less condom use during PrEP scenarios) reduced the number of infections averted in all three settings in all four PrEP scenarios (Table 1). In PrEP scenarios with both low coverage and low effectiveness, condom substitution even led to an increase in the number of HIV infections in all three settings. In Botswana and in Southern India, the impact of the high PrEP scenario targeting only sex workers was also nullified by condom substitution. The effect of the high PrEP scenario targeting both sex workers and clients was substantially reduced, but not nullified, by condom substitution.

In African settings, the effect of condom addition (i.e. 15% more condom use during PrEP scenarios) was highest in low PrEP scenarios targeting sex workers only (Table 1). Additional condom use during the low PrEP scenarios targeting both sex workers and clients more than doubled the number of averted HIV infections. Condom addition in the high PrEP scenarios resulted in 10 to 100% more averted infections.

The impact of condom addition in Southern India (i.e. 2.5% more condom use during PrEP scenarios) was high in low PrEP scenarios (80 to 300% more averted infections). In high PrEP scenarios, condom addition resulted in 3 to 20% more averted HIV infections.

## Discussion

PrEP strategies with high efficacy and high coverage can have a substantial impact in African settings. In Southern India, by contrast, the number of averted HIV infections in different PrEP scenarios would be much lower. The impact of PrEP may be strongly diminished or even reversed by behavioral disinhibition, especially in scenarios with low coverage and low effectiveness. However, additional condom use during low coverage and low effective PrEP doubled the amount of averted HIV infections.

We did not model ART treatment in the different epidemics. Since ART and PrEP could be the same drugs and ART is being scaled-up in many resource-poor countries, it is very unlikely that in reality PrEP would be introduced in an area without ART. ART coverage among HIV-infected adults in need of ART according to WHO criteria was estimated to be 79% in Botswana, 33% in Kenya and 4–9% in India by the end of 2006 (www.who.int/globalatlas/default.asp). Furthermore, we ignored the effect of PrEP on onward transmission, although an approximate 80% reduction in HIV transmission was shown in discordant couples where HIV-positive partners were taking ART [34]. PrEP users who become infected may also have reduced infectivity. Arguably, we may thus have underestimated the overall effect of PrEP use on HIV spread.

In our model, PrEP users who got HIV infected remained on PrEP on average for one year. This was done to reflect the reality that persons taking PrEP who get infected will be unaware of their changed HIV status until being tested again. Thus, in our model, condom substitution also affects such HIV-positive individuals making the effect of such substitution worse. Frequent HIV testing would moderate this adverse effect, but also put a heavy additional burden on health care resources.

We found that PrEP strategies could have a substantial impact in African settings. Another recent modeling study estimated a comparable impact of PrEP in sub-Saharan Africa [35]. In Botswana, we found 29,399 averted infections and 3,745,054 HIV-negative adult person years resulting in 785 averted infections per 100,000 person years (Table 1). The number of person years on PrEP was 216,541. Converting our result in averted infections per person year on PrEP, like Abbas *et al.* did, we found 0.14 averted infections per person year. They found 0.33 averted HIV infections in a similar PrEP scenario (i.e. 90% effectiveness, 75% coverage, targeting high-risk individuals) [35]. The difference might be explained by the different model assumptions. We modeled high-risk behavior explicitly by including sex workers and clients, who were in these compartments for a certain period (on average four years for sex workers and ten years for clients) and afterwards changed to low-risk individuals. Abbas *et al.* used four different sexual activity levels, that lasted lifelong. Moreover, they also included reduced infectivity when a PrEP user got infected with HIV, which was not in our model.

We performed sensitivity analyses of PrEP coverage in all three settings. We changed PrEP coverage in steps of 5% with both PrEP efficacy levels (50% and 90%) and both target groups (CSW only and CSWs & clients), ranging from 15–35% and 65–85% in African settings and from 40–60% and 80–99% fro Southern India. In Southern India, we also looked at low coverage varying from 15% to 35%, which is comparable to the African settings. We found that the number of averted HIV infections in the period 2012–2022 was almost proportional to the coverage of PrEP. For the interventions directed at CSW only, there was a modest additional effect with higher PrEP coverage due to a slightly reduced level of transmission within the population (results not shown).

One of the added values of our study is that we also modeled the impact of PrEP in Southern India. The number of HIV infections averted was much lower than in the African settings. This is primarily due to the high levels of condom use during commercial sex in Southern India that have resulted in a steeply decreasing HIV trend since 2000 [29]–[31]. If PrEP would even slightly decrease current condom use levels in India, its impact would be negative. Furthermore, India is a densely populated country where about 300 million people live in the Southern states at this moment (www.censusindia.gov.in). The population grows with 1.4% per year, resulting in about 370 million people in 2022. On a population level, around 2,700 HIV infections can be averted in Southern India in a 10-year PrEP scenario targeting 50% of the sex workers and with 50% effectiveness. However, if during this PrEP scenario 15% less condoms are used, this leads to an additional 51,000 HIV infections, and even 180,000 more infections if condom use goes down to 60%. Thus, in India introduction of new prevention methods such as PrEP must be done very carefully in order not to compromise the benefits gained through condom use.

Prophylaxis can be a useful method to prevent HIV infection, especially for women. However, large-scale PrEP use might encounter problems such as poor adherence and resistance. In a study in Zambia, 30% of tuberculosis patients in a Directly Observed Therapy programme stopped medication prematurely, before the completion of the scheduled 8-month treatment course [36], and adhering to pills for disease prevention might even be more difficult than for treatment. Clearly, assuring long-time adherence might be one of the tremendous difficulties facing PrEP services.

One of the problems of intermittent use, besides reduced effectivity, is possible emergence of resistant viruses. We assumed that PrEP use would not lead to resistance to ART drugs, based on the modeling work of Smith *et al* for a clinical trial situation in Botswana (2006, THAX0105–Antiretroviral resistance is not an important risk of the oral tenofovir prophylaxis trial in Botswana: a simple mathematical modelling approach, XVI International AIDS Conference, Toronto, Canada). PrEP use on a wider scale outside trial settings with more people taking PrEP, a higher risk of non-adherence, and possible changes in risk behavior due to less extensive counseling may well lead to the emergence of resistant strains. Although usage of PrEP pills that contain two or more different ARV drugs may decrease the risk of development of resistance, it may not ultimately prevent it.

We have demonstrated that disinhibition during PrEP services is important and may have a considerable effect on HIV epidemics. To what extent disinhibition will actually occur is still uncertain. Earlier studies of the effect of PEP or ART on disinhibition reported conflicting results. Studies of PEP in homosexual men in the US did not demonstrate an increase in risky sexual behavior [37], [38]. Similarly, a meta-analysis on sexual behavior and ART in industrialized countries did not reveal an increase in risky behavior of persons receiving ART compared to those who did not, except in those who believed that therapy prevented transmission [39]. Providing ART and counseling was even associated with reduced sexual risk behavior in Uganda [40]. In South Africa, by contrast, high levels of unprotected sex were reported both by persons on ART and by those not yet eligible for ART [41].

Condom addition had a substantial impact on the number of averted HIV infections in the different PrEP scenarios. People coming for HIV-testing and PrEP should be extensively counseled about the necessity to continue or enhance habits of safe sex, such as use of condoms. Furthermore, PrEP pills could be distributed in combination with condoms. Changes in risk behavior can be assessed by repeated sexual behavior surveys or by STD screening when people visit clinics for scheduled PrEP pills collections.

We conclude that PrEP can have a substantial impact in the reduction of HIV. Targeting high-risk groups is relatively easy and inexpensive and would result in comparable HIV prevalences as successful condom interventions. However, policy makers should be aware of changes in risk behavior. Especially in Southern India, where condom use is already very high during commercial sex, small changes could have strong negative effects. Implementation of PrEP strategies should come on top of current condom campaigns, not as a substitution.

## Supporting Information

Appendix S1 Formal structure of the model
(0.06 MB DOC)

Figure S1 (4.40 MB DOC)
