# Supplementary material for: The Impact of Pre-Exposure Prophylaxis (PrEP) on HIV Epidemics in Africa and India: A Simulation Study
Source: PLoS One. 2008 May 7;3(5):e2077. doi: 10.1371/journal.pone.0002077 (PMC2367053; doi:10.1371/journal.pone.0002077)
Supplement: Appendix S1 — Formal structure of the model (0.06 MB DOC) [file pone.0002077.s001.doc]

**Appendix S1: Formal structure of the model**

A schematic overview of the model can be found in Figure S1. Boxes represent compartments, i.e. the states males or females can be in. Arrows represent flows of individuals between compartments. High-risk groups are male clients and female sex workers. Disease progression is subdivided into 2 stages: early and late, including AIDS. Individuals move to the PrEP box when PrEP is initiated. Symbols refer to compartments and flows formally defined below.

Names of compartments and flows were chosen as follows. Compartments: G = gender, male or female; i = first subscript with 1 = low risk group, 2 = high risk group; j = second subscript with 1 = uninfected, 2 = early HIV, 3 = late HIV, 4 = taking PrEP and uninfected, 5 = taking PrEP and early HIV. Flows: a = from low-risk to high-risk group, b = from high-risk to low-risk group, t = start taking PrEP, u = stop taking PrEP, i = infection, p = progression (to late stage HIV infection), q = death.

PrEP use can be modelled in the general population and in high-risk groups. Only the latter was used in the main article. Parameters names are in *italics,* variable names are in normal font. Parameter values that are identical for all three settings are only shown once.

**COMPARTMENTS**

| **Symbol Figure 1** | **Equation** |
| --- | --- |
| G_11_ | In women: -*mu_neg**G_11_ - aG_11_ + bG_21_ + bG_24_ - tG_11_ + uG_14_ - iG_11_ + population**femgr*  In men: -*mu_neg**G_11_ - aG_11_ + bG_21_ + bG_24_- tG_11_ + uG_14_ - iG_11_ + population**malegr* |
| G_12_ | *-mu_pos**G_12_ - aG_12_ + bG_22_ + uG_15_+ iG_11_ - pG_12_ |
| G_13_ | *-mu_pos******G_13_ **-** aG_13_ + bG_23_ + pG_12_ + pG_15_- qG_13_ |
| G_14_ | *-mu_neg**G_14_ - aG_14_ + tG_11_ - uG_14_- iG_14_ |
| G_15_ | *-mu_pos**G_15_ - aG_15_ + bG_25_ - uG_15_ + iG_14_ - pG_15_ |
| G_21_ | *-mu_neg**G_21_ + aG_11_ - bG_21_ - tG_21_ + uG_24_ - iG_21_ |
| G_22_ | *-mu_pos**G_22_ + aG_12_ - bG_22_ + uG_25_+ iG_21_ - pG_22_ |
| G_23_ | *-mu_pos******G_23_ **+** aG_13_ - bG_23_ + pG_22_ + pG_25_- qG_23_ |
| G_24_ | *-mu_neg**G_24_ + aG_14_ - bG_24_ + tG2_1_ - uG_24_ - iG_24_ |
| G_25_ | *-mu_pos**G_25_ + aG_15_ - bG_25_ - uG_25_ + iG_24_ - pG_25_ |
| AIDS death | qG_13_ + qG_23_ |

FLOWS (transitions between compartments)

| **Symbol Figure 1** | **Equation** |
| --- | --- |
| aG_1j_ (j=1,…,5) | In women: *prof* * G_1j_* exp (annualCSWcontacts / *mkt* - 1)  In men: *cust* * G_1j_ |
| bG_2j_ (j=1,…,5) | In women: *unprof* * G_2j_  In men: *uncust* * G_2j_ |
| iG_11_ | In women: leakmen * (G_12_ + G_13_ + G_15_ + G_22_+ G_23_ + G_25_) * G_11_ / non_csw + G_11_ * *stabfactor* * *mf_risk* * marrate_female * (G_12_ + G_13_ + G_15_) / non_clients  In men: leakwomen * (G_12_ + G_13_ + G_15_ + G_22_ + G_23_ + G_25_) * G_11_ / non_clients + G_11_ * *stabfactor* * *fm_risk* * *marrate_male* * (G_12_ + G_13_ + G_15_) / non_csw |
| iG_14_ | In women: (1 – *PrEP_efficacy*) * (leakmen * (G_12_ + G_13_ + G_15_ + G_22_ + G_23_ + G_25_) * G_14_ / non_csw + G_14_ * *stabfactor* * *mf_risk* * marrate_female * (G_12_ + G_13_ + G_15_) / non_clients)  In men: (1 – *PrEP_efficacy*) * (leakwomen * (G_12_ + G_13_ + G_15_ + G_22_ + G_23_ + G_25_) * G_14_ / non_clients + G_14_ * *stabfactor* * *fm_risk* * *marrate_male* * (G_12_ + G_13_ + G_15_) / non_csw) |
| iG_21_ | In women: G_21_ * annualCSWcontacts * *mf_risk* * (1 – *condom_prot*) * (G_22_ + G_23_ + G_25_) / clients  In men: G_21_ * *cont_rate* * *fm_risk* * (1 – *condom_prot*) * (G_22_ + G_23_ + G_25_) / csw |
| iG_24_ | In women: (1 – *PrEP_efficacy*) * G_24_ * annualCSWcontacts * *mf_risk* * (1 – *condom_prot*) * (G_22_ + G_23_ + G_25_) / clients  In men: (1 – *PrEP_efficacy*) * G_24_ * *cont_rate* * *fm_risk* * (1 – *condom_prot*) * (G_22_ + G_23_ + G_25_) / csw |
| pG_ij_ (i=1,2; j=2,5) | *hivprog* * G_ij_ |
| qG_i3_ (i=1,2) | *mu_aids* * G_i3_ |
| tG_i1_ (i=1,2) | *PrEP_rate* * G_i1_ |
| uG_ij_ (i=1,2; j=4) | *rate_stop_PrEP* * G_ij_ |
| uG_ij_ (i=1,2; j=5) | *rate_stop_PrEP_early* * G_ij_ |

### VARIABLES

| **Variable Name** | **Defining equation** |
| --- | --- |
| non_csw or non_clients | G_11_ + G_12_ + G_13_ + G_14_ + G_15_ |
| csw or clients | G_21_ + G_22_ + G_23_ + G_24_ + G_25_ |
| females | non_csw + csw |
| males | non_clients + clients |
| population | females + males |
| annualCSWcontacts | *cont_rate* * clients / csw |
| leakwomen | *leak* * 2 * *fm_risk* / (*fm_risk* + *mf_risk)* |
| leakmen | *leak* * 2 * *mf_risk* / (*fm_risk* + *mf_risk)* |
| marrate_female | *marrate_male* * non_clients / non_csw |
| female_prevalence | (females - G_11_ - G_21_- G_14_ - G_24_) / females |
| male_prevalence | (males - G_11_ - G_21_ - G_14_ - G_24_) / males |
| uninf_on_PrEP | G_14_ + G_24_ |
| HIV_on_PrEP | G_15_ + G_25_ |
| total_on_PrEP | uninf_on_PrEP + HIV_on_PrEP |

**MODEL PARAMETERS (values)**

Values that are identical for all settings are only shown once.

| **Parameter name** | **Parameter description / interpretation** | **Botswana** | **Nyanza province** | **Southern India** |
| --- | --- | --- | --- | --- |
| *femgr* | Annual growth rate adult female population | 0.04 | | |
| *malegr* | Annual growth rate adult male population | 0.04 | | |
| *mu_neg* | Annual mortality rate HIV negatives | 0.026 | | |
| *mu_pos* | Annual mortality rate (non-AIDS) HIV positives | 0.028 | | |
| *mu_aids* | Annual rate of AIDS death among late stage HIV infected | 1 | | |
| *fm_risk* | Probability of female-to-male transmission per high-risk contact | 0.0125 | | |
| *mf_risk* | Probability of male-to-female transmission per high-risk contact | 0.03 | | |
| *hivprog* | Annual rate of developing late stage HIV among early stage HIV infected | 0.25 | | |
| *marrate_male* | Annual rate of establishing stable relationships (men) | 0.232 | | |
| *stabfactor* | Multiplier for stable relationships | 25 | | |
| *leak* | Annual HIV transmission to non-commercial and non-marital partners | 0.11 | 0.065 | 0.04 |
| *cust* | Annual rate of becoming CSW client | 0.025 | | |
| *uncust* | Annual rate of becoming low-risk male among clients | 0.1 | | |
| *prof* | Annual rate of becoming CSW, when CSW have *mkt* clients annually | 0.025 | | |
| *unprof* | Annual rate of becoming low risk female among CSW | 0.25 | | |
| *cont_rate* | Annual number of CSW contacts per client (rate) | 26 | | |
| *mkt* | Parameter controlling the rate of becoming CSW in response to demand | 1000 | | |
| *condom_prot* | Effective condom use (%) in CSW client contacts at different time points | 20%, 50% | | 60%, 90% |
| *condom_after* | Effective condom use (%) in CSW client contacts during condom intervention | 75% | | 95% |
| *PrEP_efficacy* | Level of protection by PrEP:  Low scenarios (50%)  High scenarios (90%) | 0.5  0.9 | | |
| *PrEP_rate* | Annual rate of starting PrEP to obtain correct coverage:  Low scenarios (25% or 50%)  High scenarios (75% or 95%) | 0.22  1.1 | | 0.37  6.0 |
| *rate_stop_PrEP* | Annual rate of stopping PrEP if uninfected | 0.04 | | |
| *rate_stop_PrEP_early* | Annual rate of stopping PrEP if HIV early | 1 | | |
